# Postoperative periprosthetic fractures associated with compressive osseointegration: a systematic review of the Zimmer Biomet Compress^®^ device

**DOI:** 10.1007/s00402-026-06477-z

**Published:** 2026-08-22

**Authors:** Kazuhiko Hashimoto, Shunji Nishimura, Koji Goto

**Affiliations:** Department of Orthopedic Surgery, Kindai Univeristy Hospital, Sakai, Japan

**Keywords:** Compressive osseointegration, Periprosthetic fracture, Compress device, Limb salvage, Endoprosthetic reconstruction

## Abstract

The Zimmer Biomet Compress^®^ Compliant Pre-Stress (CPS) device uses a compressive osseointegration technique for endoprosthetic fixation and provides an alternative to stemed implants. Despite having benefits, such as bone stock retention, periprosthetic fractures continue to remain a concern. Therefore, this systematic review presents the prevalence, risk factors, and outcomes of the CPS device and periprosthetic fractures after treatment. Medical databases (PubMed, Embase, and Google Scholar) were searched to identify studies reporting complications related to the CPS device. Peer-reviewed studies with at least 2 years of follow-up for fracture rates, revision results, and implant survivorship were included. The primary data collected were fracture incidence and timing of complications, and the CPS device was compared with the traditional cemented stem. This review highlights important studies, including a cohort of 221 patients and pediatric data from 36 patients. Adult populations showed a periprosthetic fracture rate of 2.7–3.9%. A distinct temporal pattern emerged: the frequency of fractures was most pronounced within the first 2 years after surgery. In the pediatric context, spindle survivorship was 86.3% at 5–10 years, despite higher rates of mechanical complications. Notably, for most fracture cases, the bone–implant interface was radiographically stable, providing easier revision with significantly less bone loss with the CPS device than with the traditional cemented stem. The CPS device showed a periprosthetic fracture rate similar to or lower than that of the traditional cemented stem and an early onset temporal profile with distinct features. The stability of the bone–implant interface despite fracture and its ability to be modified after implantation highlight its suitability for limb salvage surgery. To reduce the fracture risk, strict postoperative weight-bearing protocols are recommended during the early osseointegration period. This review highlights important studies, including a cohort of 221 patients and pediatric data from 36 patients. Adult populations showed a periprosthetic fracture rate of 2.7–3.9%. A distinct temporal pattern was observed, with fractures occurring most frequently within the first 2 years after surgery. In pediatric populations, spindle survivorship was 86.3% at 5 years and 66.2% at 10 years, despite higher rates of mechanical complications. Notably, for most fracture cases, the bone–implant interface remained radiographically stable, providing easier revision with significantly less bone loss than with traditional cemented stems. The CPS device showed low reported adult periprosthetic fracture rates and an early-onset temporal profile with distinct features. The stability of the bone–implant interface despite fracture occurrence, together with the ability to modify the implant after implantation, highlight its suitability for limb salvage surgery. To reduce fracture risk, strict postoperative weight-bearing protocols are recommended during the early osseointegration period.

## Introduction

**Fig. 1:**
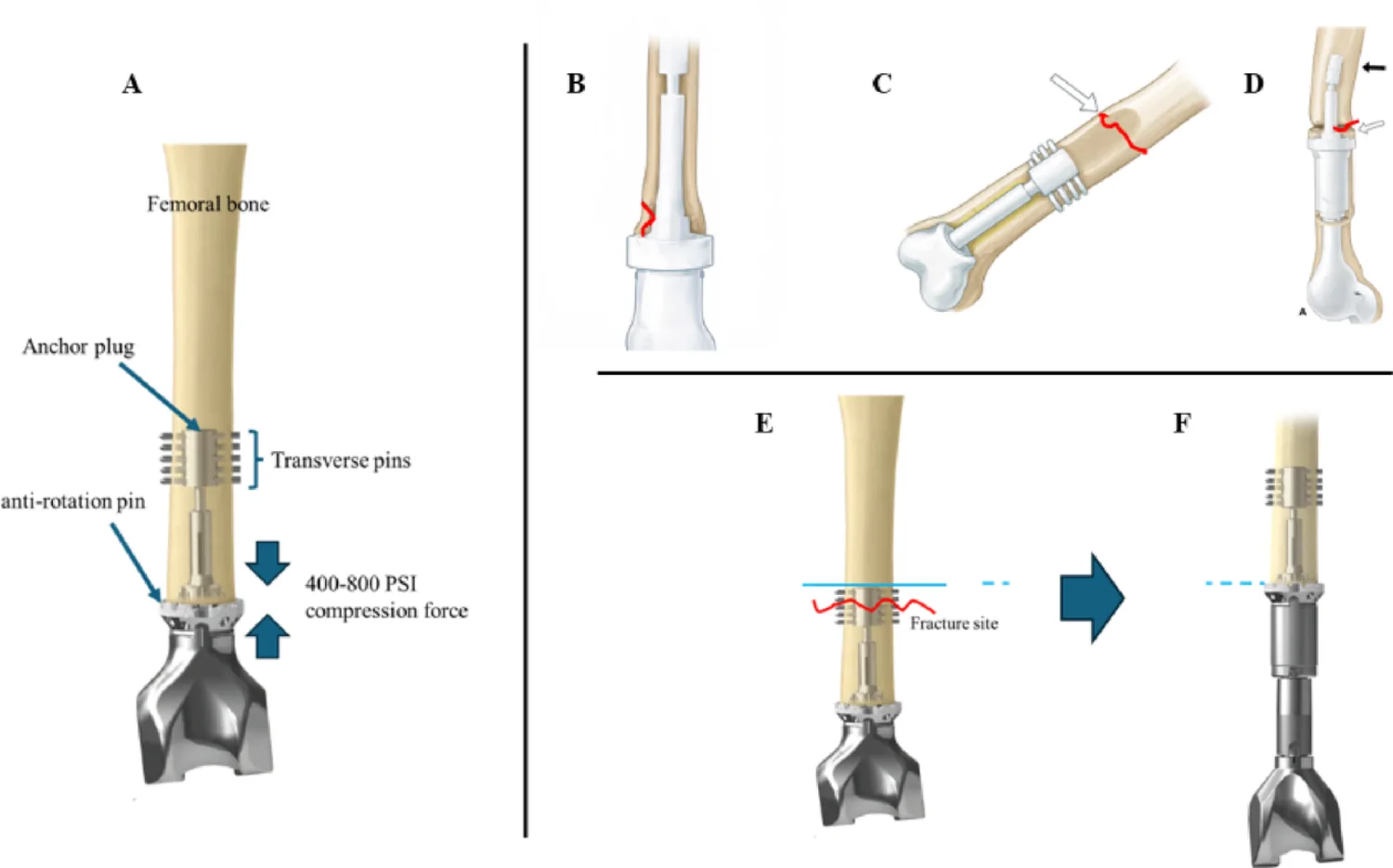
Compress^®^ Device: Mechanism and Failure Classification. Anatomically accurate schematic representation of the compressive osseointegration mechanism of the Compress^®^ device and failure modes based on Healey et al.’s classification [3]. **A** Cross-sectional view of the Compress^®^ device properly seated in the distal femur. The short intramedullary anchor plug (total length, approximately 80 mm) is fixed by three transverse pins penetrating both cortices. The Belleville washer stack within the spindle generates 400–800 PSI (181–363 kg) of continuous compression at the bone–implant interface. This compressive force promotes cortical hypertrophy and osseointegration while avoiding stress shielding. The spindle length ranges from 45–80 mm depending on bone diameter. **B–D** Lateral views of the distal femur illustrating the two primary periprosthetic fracture patterns observed in clinical series [1]. **B** Type I (interface failure with fracture), arising between the spindle and anchor plug. **C** Type IIA (proximal fracture), arising proximal to the anchor plug. **D** Type IIB (posterior cortical avulsion fracture), arising at the posterior cortex of the spindle. **E**,** F** Surgical management of post-implant fractures. E: Schematic illustration of the fracture; the red wavy line indicates the fracture line, and the blue solid line denotes the planned osteotomy for revision surgery. **F** Schematic illustration of the reconstructive procedure after fracture; the blue dotted line indicates the osteotomy site, and the resultant length deficiency is bridged with a spacer during reconstruction

Massive endoprosthetic (EPP) reconstruction after oncological resection or complex trauma has raised critical concerns regarding implant fixation and longevity [1, 2]. Traditional long-stemmed cemented or press-fit implants are the gold standard of care but are associated with stress shielding and aseptic loosening, which can lead to challenging revision scenarios requiring substantial bone loss is required [3, 4]. The Zimmer Biomet Compress^®^ Compliant Pre-Stress (CPS) device uses the Wolff law to maintain bone stock through compressive osseointegration, allowing a major departure in fixation technology [5, 6]. The CPS device gains fixation through a short intramedullary traction bar and spring-loaded spindle with a high compressive force at the bone–implant interface of approximately 400–800 pounds [7] (Fig. 1A-F). This mechanism is designed to protect against stress shielding and to stimulate cortical bone hypertrophy [8, 9]. The device comprises three central modules: an anchor plug fixed with transverse pins, a short spindle containing Belleville washers that provide compression, and an adaptor attached to the modular endoprosthetic components. Despite their biomechanical benefits, the high compressive forces generated at the implants increase the risk of potential periprosthetic fractures, particularly at the anchor plug site and the bone–implant interface [10]. Knowledge of the epidemiology and failure mechanisms of these devices is important for surgical decision making. Compressive osseointegration devices exhibit a failure profile distinct from that of cemented stems [11, 12]; the latter fails because of late-onset aseptic loosening and osteolysis [13, 14]. Therefore, this systematic review aimed to comprehensively evaluate the occurrence, timing, and management of postoperative periprosthetic fractures associated with the CPS device and compare these outcomes with those of the traditional cemented stem.

## Materials and methods

### Study design and literature search

**Fig. 2:**
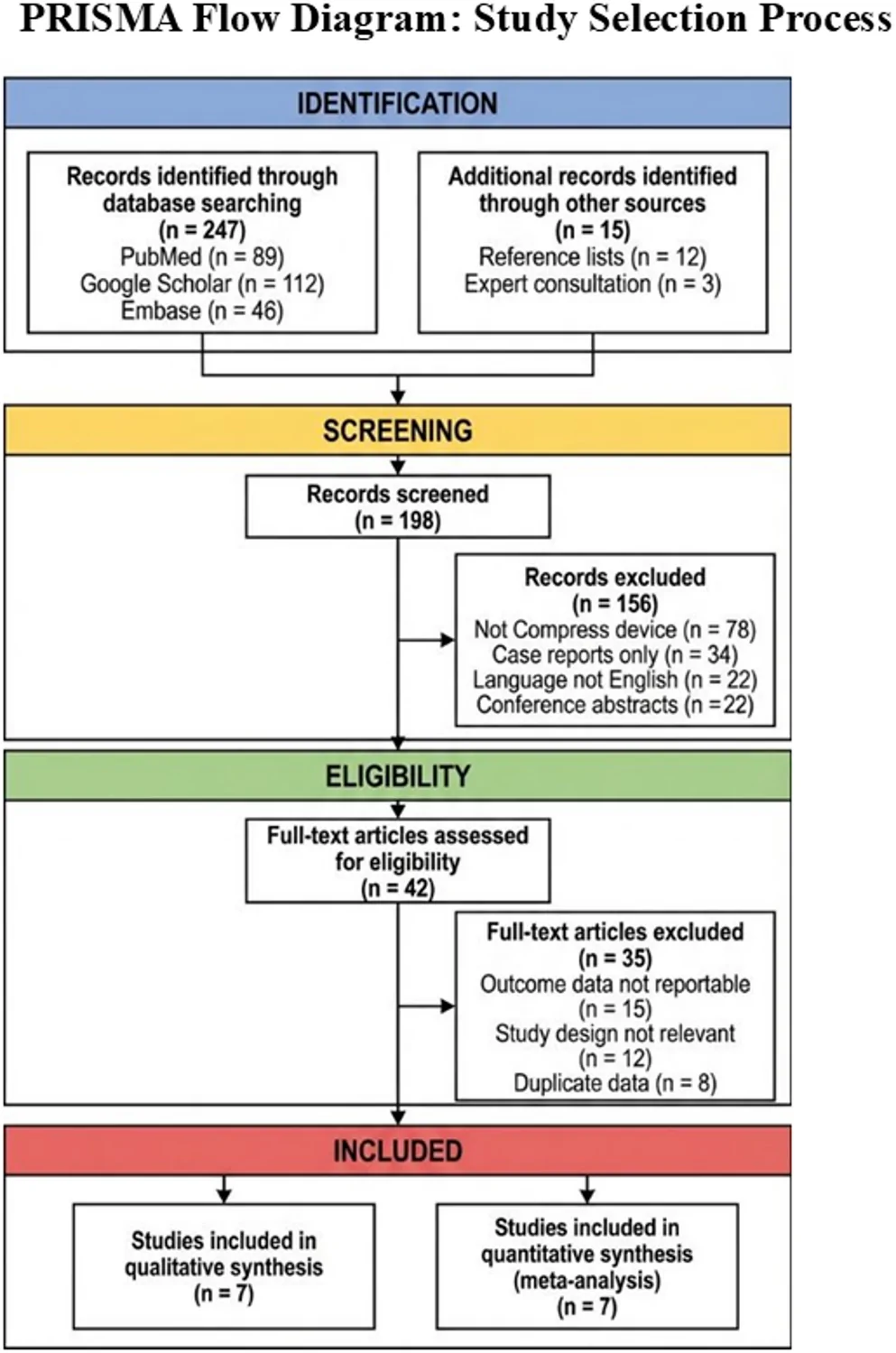
PRISMA Flow Diagram: Study Selection Process. Preferred Reporting Items for Systematic Reviews and Meta-Analyses (PRISMA) flow diagram illustrating the study selection process. The initial search identified 247 records through database searching (PubMed *n* = 89, Google Scholar *n* = 112, Embase *n* = 46) and 15 additional records through other sources (reference lists *n* = 12, expert consultation *n* = 3). After removing duplicates, 198 records were screened. Following exclusion of studies not involving the Compress^®^ device (*n* = 78), case reports only (*n* = 34), non-English-language publications (*n* = 22), and conference abstracts (*n* = 22), 42 full-text articles were assessed for eligibility. Of these, 35 were excluded owing to non-reportable outcome data (*n* = 15), irrelevant study design (*n* = 12), or duplicate data (*n* = 8). Seven studies met inclusion criteria and were included in qualitative and quantitative syntheses (meta-analysis)

This systematic review was conducted in accordance with the Preferred Reporting Items for Systematic Reviews and Meta-Analyses guidelines [15] (Fig. 2). An electronic search was performed in the PubMed/MEDLINE, Embase, and Google Scholar databases from inception to December 2024. The search terms included “Zimmer Biomet Compress,” “Compressive Osseointegration,” “Periprosthetic Fracture,” “Postoperative Complications,” and “Limb Salvage” [16].

### Inclusion and exclusion criteria

Studies were included **if** they were published in peer-reviewed English-language journals; involved human participants treated with the CPS device for oncologic, traumatic, or revision indications; and reported specific data on postoperative fracture rates or implant survivorship with a minimum follow-up of 2 years [17]. Case reports with fewer than five patients and biomechanical studies without clinical data were excluded [18].

### Data extraction and analysis

Data regarding patient demographics, surgical indications, follow-up duration, incidence of periprosthetic fractures, timing of fractures relative to the index surgery, status of the osseointegration interface at the time of failure, and revision strategies were extracted [19]. Comparative data on traditional cemented stems were also aggregated for analysis from relevant studies [20, 21].

### Methodological quality assessment

Because all included studies were non-randomized observational studies, methodological quality was assessed using the Methodological Index for Non-Randomized Studies (MINORS). The eight-item version was used for non-comparative studies and the 12-item version for comparative studies, as appropriate. Two reviewers independently performed the assessment, and disagreements were resolved through discussion and consensus.

The authors used the generative AI tool GenSpark to assist in developing and refining the literature search strategy for this systematic review. All database searches, screening decisions, data extraction, and data synthesis were performed manually by the authors. The authors reviewed and verified all AI-assisted outputs and take full responsibility for the accuracy and integrity of the final review.

## Results

### Literature search

The search strategy yielded 127 potentially relevant studies, of which seven studies met the inclusion criteria for detailed analysis. Primary data were derived from the cohorts reported by Tyler et al. [11], Healey et al. [12], Tanaka et al. [22], Goldman et al. [23], Corona et al. [24], Monument et al. [25], and Kagan et al. [26]. Across these seven cohorts, 455 cases were tumor-related/oncologic and 109 were non-oncologic, based on the indication categories reported in the original studies.

### Methodological quality of the included studies

Overall, the included studies demonstrated moderate methodological quality. Common limitations included retrospective study design, lack of prospective sample size calculation, absence of blinded endpoint assessment, and limited availability of appropriate control groups. These methodological constraints should be considered when interpreting the reported fracture rates, survivorship outcomes, and risk factor analyses.

### Incidence of periprosthetic fractures

The total number of periprosthetic fractures or aseptic mechanical failures related to the CPS device was generally low across studies (Table 2). Tyler et al. [11] conducted a large multicenter cohort study of 221 patients and reported a periprosthetic fracture rate of 2.7% (*n* = 6); among distal femoral implants, the rate was 3.9% (6/154). The reported fracture rate appears low in absolute terms. However, direct comparison with the cumulative risk of aseptic loosening reported for cemented megaprostheses should be interpreted cautiously because follow-up durations and failure endpoints differ among studies [27, 28] (Table 1). Monument et al. [25] reported one periprosthetic fracture (4.5%) in a femoral oncologic cohort of 22 patients, whereas Kagan et al. [26] reported an overall failure rate of 22%, with aseptic mechanical failure accounting for 5.3% (*n* = 6) of 114 cases. Goldman et al. [23] reported a 9% aseptic mechanical failure rate, with no additional aseptic mechanical failures after 2 years.

**Table 1:** Key data points from the selected studies

Study	*N*	Population	Follow-up (months)	Periprosthetic fracture/mechanical rate	Key findings
Tyler et al. (2009) [11]	221	Adult patients with compressive osseointegration reconstruction, including primary oncology 165, revision oncology 33, revision arthroplasty 18, and post-traumatic 5	53 (mean)	2.7% (*n* = 6)	All fractures occurred within 2 years, and the bone-implant interface remained stable in all fracture cases.
Healey et al. (2013) [12]	82	Adult patients undergoing Compress knee arthroplasty, including tumor reconstruction 80 and noncancer revision TKA 2	60 (median)	Approximately 3%	Ten-year all-cause survivorship was 80%, with high interface stability and minimal aseptic loosening.
Tanaka et al. (2023) [22]	36	Pediatric oncologic patients, including osteosarcoma 34 and Ewing sarcoma 2	87.4 (mean)	18% (*n* = 6)	Spindle survivorship was 86.3% at 5 years and 66.2% at 10 years, and the complication rate was relatively high because of expandable components.
Goldman et al. (2016) [23]	79	Adult distal femur oncologic reconstruction cases	60 (mean)	9% (aseptic mechanical failure)	Spindle survival free from mechanical failure was 91% at 5 and 10 years, and no aseptic mechanical failures occurred after 2 years.
Corona et al. (2021) [24]	10	Adult non-oncologic infected post-traumatic distal femur defects	27 (median)	Not separately reported	Limb salvage was achieved in all cases, with no recurrence of infection during follow-up.
Monument et al. (2015) [25]	22	Femoral oncologic cohort, including distal femur 19 and proximal femur 3	Minimum 60	4.5% (*n* = 1)	Five-year survivorship free from aseptic failure was 89%.
Kagan et al. (2017) [26]	114	Lower extremity reconstruction cases, including primary oncologic 40, revision arthroplasty 69, and fracture 5	41 (mean)	5.3% (*n* = 6; aseptic mechanical failure)	Overall failure rate was 22%, largely due to infection and tumor; aseptic mechanical failure accounted for 5.3% of cases.

Across the seven cohorts included in the final systematic review, 455 cases were tumor-related/oncologic and 109 were non-oncologic, based on the indication categories reported in the original studies

*TKA* total knee arthroplasty

**Table 2:** Comparison of the compress^®^ compliant pre-stress device versus the traditional cemented stem

Feature	Compress^®^ compliant pre-stress device	Traditional cemented stem
Primary failure mode	Early periprosthetic fracture/Lack of osseointegration	Late aseptic loosening/Osteolysis
Timing of failure	< 2 years (Early)	> 5–10 years (Late, progressive)
Fracture rate	2.7–3.9%	Variable (associated with osteolysis)
Aseptic loosening rate	5–11% (at 5 years)	12–15% (at 10 years)
Bone stock preservation	High (Minimal resection for revision)	Low (Significant bone loss during removal)
Revision complexity	Low to Moderate	High
Weight-bearing protocol	Protected for 6–12 weeks	Immediate to early

### Temporal pattern of failure

**Fig. 3:**
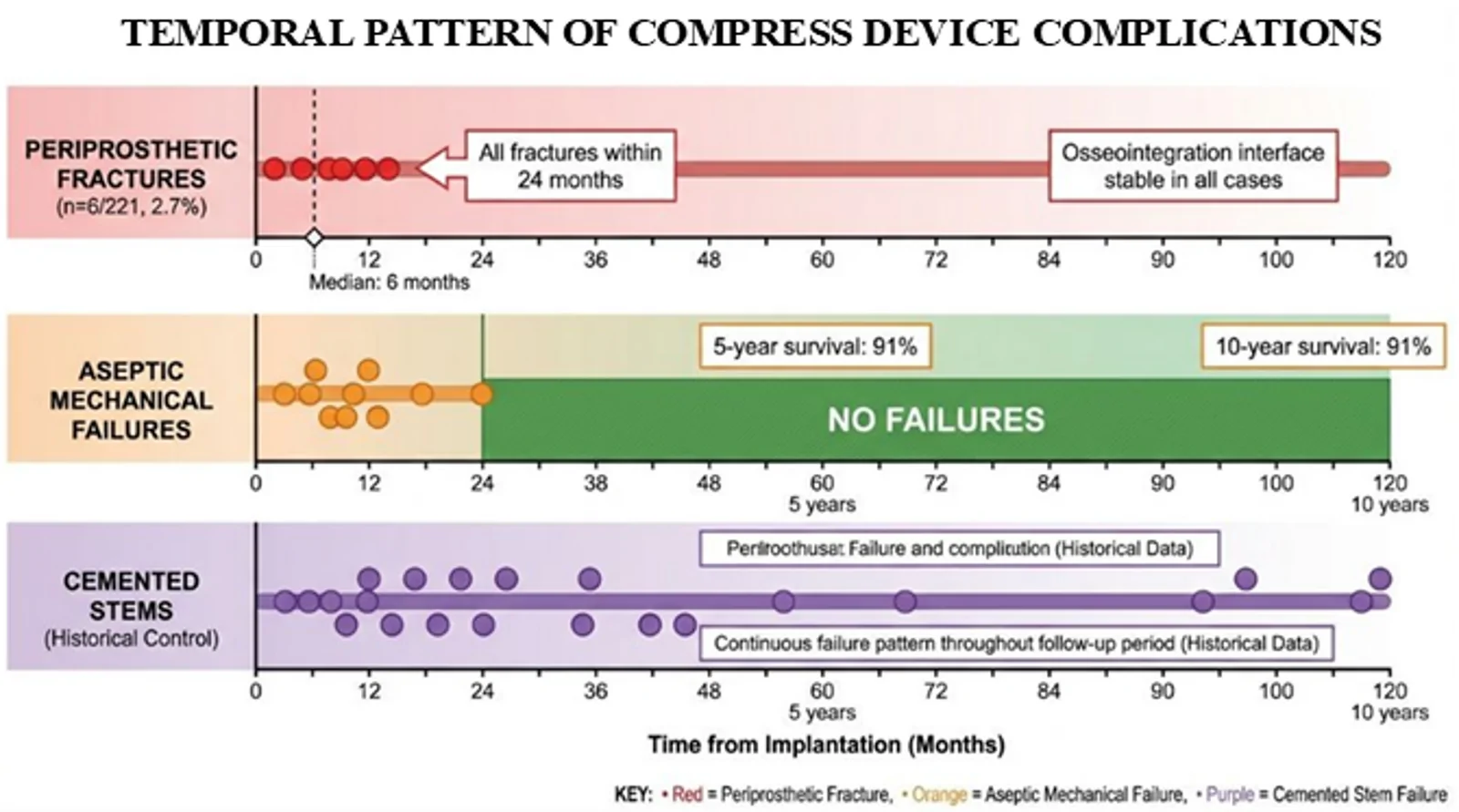
Temporal Pattern of Complications Associated with the Compress^®^ Device. Temporal distribution of complications between the Compress^®^ device and traditional cemented stem. Top Panel (Red): Periprosthetic fractures (*n* = 6/221, 2.7%) occurred exclusively within the first 24 months post-implantation (median: 6 months), with all cases maintaining stable osseointegration interfaces despite fracture. Middle Panel (Orange/Green): Aseptic mechanical failures clustered within the first 24 months, after which no additional failures occurred, resulting in 91% survivorship at 5 and 10 years. The green zone represents a “no failures” period after successful osseointegration. Bottom Panel (Purple): Historical control data from cemented stems showing a continuous, progressive failure pattern throughout the follow-up period, contrasting sharply with the early-onset, time-limited failure pattern of the Compress^®^ device. This unique temporal profile suggests that the critical period for complications associated with the Compress^®^ device is during initial osseointegration (0–24 months), after which the construct demonstrates excellent durability. Key: Red dots = periprosthetic fracture events; orange dots = aseptic mechanical failure events; purple dots = cemented stem failure events (historical data)

A consistent temporal pattern of events was observed across all studies (Table 1; Fig. 3). Unlike the traditional cemented stem, where the risk of failure increases with over time owing to wear debris and osteolysis [29, 30], periprosthetic fractures with the CPS device occur primarily within 2 years after surgery. In the series by Tyler et al. [11], the median time to fracture was 6 months, with a range of 2–20 months. All six periprosthetic fractures occurred within 24 months of implantation, and the osseointegration interface remained stable in all cases (Fig. 3). Goldman et al. [23] explicitly stated that spindle failures only occurred in the first 2 years, with no mechanical failures thereafter. This temporal distribution suggests that once osseointegration and cortical hypertrophy were achieved, the construct becomes mechanically robust. Healey et al. [12] supported this observation and stated that most mechanical complications occurred during the first 18 months, after which the implant demonstrated excellent survivorship. As shown in Fig. 3, aseptic mechanical failures were commonly clustered during the early postoperative period. The 5-year and 10-year survival rates remained constant at 91%, suggesting that no further late mechanical failures occurred after the critical osseointegration period.

### Pediatric outcomes

High activity levels and skeletal immaturity contribute to unique challenges in the pediatric population. Tanaka et al. [22] included 36 pediatric patients who received distal femoral CPS devices with expandable components, with a mean follow-up duration of 87.4 months. Although the overall mechanical complication rate was high (81% experienced some type of International Society of Limb Salvage failure) because of the complexity of the expandable components, spindle survivorship was 86.3% at 5 years and 66.2% at 10 years. Periprosthetic fractures occurred in 16.7% (6/36) of pediatric patients, with a mean time to fracture of 5.7 years [22]. These findings suggest that greater activity levels and prolonged skeletal remodeling in pediatric patients may contribute to a different fracture risk profile than that observed in adults.

### Interface stability during fracture events

Another important conclusion drawn from the reviewed studies is that the stability of the bone–implant interface during fracture events is key. Tyler et al. [11] noted that the osseointegrated interface remained radiographically stable in all six periprosthetic fractures. The most common fracture pattern occurred above the anchor plug (five of six cases), with only one fracture at the anti-rotation pin insertion site. This stability offers less aggressive revision protocols than those required for failed traditional cemented stems, which often require extended osteotomies or cortical windowing to remove cement [31, 32]. Regarding infected reconstructions, Corona et al. [24] noted that despite difficult cases of bone loss and infection, preservation of the compression interface can be accomplished during revision. Preserving an osseointegrated interface preserves the bone stock and makes revision surgery more accessible. Generally, as illustrated in Fig. 1, the revision plan consists of stem extension with plating or a two-stage strategy in cases of infection, with the goal of restoring function and maintaining the osseointegration.

### Survivorship data across studies

Table 3 summarizes the survivorship data across major studies. Overall survivorship from aseptic mechanical failure ranged from 85 to 95% at 5 years across studies, which compares favorably with historical data for the traditional cemented stem [33, 34]. Monument et al. [25] reported 89% survivorship of aseptic failure at 5 years, and Zimel et al. [35] demonstrated 89% survivorship at 5 and 10 years, suggesting that the failure rate plateaued after the initial 2-year risk period.

**Table 3:** Survivorship data across the studies

Study	Follow-up period	Overall survival	Aseptic mechanical failure	Spindle survival	Notes
Tyler et al. [11]	4.2 years (mean)	Not reported	2.7% fracture rate	97.3%	Early series, learning curve
Healey et al. [12]	5 years	85%	15%	85%	At 10 years: 80% survival
Monument et al. [27]	5 years	89%	11%	89%	Femoral locations only
Goldman et al. [23]	5–10 years	91%	9%	91%	Distal femur specific
Zimel et al. [35]	10 years	89%	11%	89%	Revision series
Tanaka et al. [22]	5–10 years	86.3%	13.7%*	86.3%	Spindle-specific survival

### Risk factor analysis

Available evidence regarding risk factors was limited and heterogeneous; however, several factors were repeatedly implicated across studies (Table 4). Traditional demographic variables such as age, sex, and body mass index, were not consistently associated with fracture risk. In contrast, device- and treatment-related factors appeared to be more relevant, including cortical thickness < 2.5 mm, early weight bearing before osseointegration, limited surgeon experience, active chemotherapy, planned radiation therapy, high activity levels, and distal femoral reconstruction.

**Table 4:** Risk factors for periprosthetic fracture

Risk factor	Risk level	Evidence quality	References	Notes
Cortical thickness < 2.5 mm	High	Strong	[12]	Manufacturer contraindication
Early weight bearing	Moderate	Moderate	[1]	Within the first 6–12 weeks
Surgeon experience < 5 cases	Moderate	Limited	[28]	Learning curve effect
Age > 70 years	Low	Conflicting	[27, 28]	Not consistently demonstrated
Chemotherapy (active)	Low	Weak	[40]	May delay but not prevent osseointegration
Radiation therapy (planned)	Moderate	Limited	[28]	Relative contraindication
High activity level	Low-Moderate	Limited	[2]	Mainly in pediatric populations
Anatomic location	Variable	Moderate	[1, 4]	Distal femur may have higher rates

## Discussion

The results of this systematic review suggest that the Zimmer Biomet CPS device may represent a useful alternative to traditional cemented stems, particularly with respect to the pattern and management of mechanical failure. In adult cohorts, reported periprosthetic fracture rates were generally low; however, direct comparisons with the long-term aseptic loosening rates of cemented stems should be interpreted cautiously because follow-up duration, patient populations, and failure endpoints differ across studies [36, 37].

### Clinical implications of the temporal failure pattern

The incidence of fractures during the first 2 postoperative years has substantial clinical implications [11, 23]. It emphasizes the importance of the early osseointegration phase. The biomechanical stability of the CPS device relies on the hypertrophy of the host bone in response to a compressive load [38]. In the first few months before hypertrophy is achieved, the construct is at risk of peak loads or torsional forces [39]. This trend highlights the need to adhere to protected weight-bearing protocols during the early postoperative period. Avedian et al. [40] demonstrated that chemotherapy can delay the initial osseointegration, potentially extending the vulnerable period. However, once osseointegration is achieved, as evidenced by cortical hypertrophy on radiographs [41], the construct demonstrates excellent durability.

### Ease of revision and bone stock preservation

One of the key benefits identified is the potential of the device to provide a “fail-safe” mechanism. When failure occurs, loss of the implant itself is rare; instead; a fractures typically occur in the bone proximal to the anchor plug [11]. Revision does not require the removal of a long intramedullary stem because the device uses a short intramedullary footprint (approximately 4–8 cm) [42]. Surgeons may resect the fractured segment and re-implant a CPS device or convert it into a stemmed implant with ample remaining bone stock. This contrasts sharply with the “catastrophic” failure mode of cemented stems, whereby extensive osteolysis causes a hollow shell of bone to form, leading to arduous reconstruction [43, 44]. Compression revision requires a mean bone resection of less than 3–4 mm on average, in contrast to the potential for extensive bone loss with stemmed implant removal.

### Risk factor analysis

Table 4 summarizes the identified risk factors of periprosthetic fractures. Interestingly, traditional demographic risk factors such as age, body mass index, and sex have not been consistently associated with an increased fracture risk [25, 26]. However, certain device-specific and surgical factors are also relevant: (1) cortical thickness < 2.5 mm represents a relative contraindication [7]; (2) limited surgeon experience < 5 cases) may be associated with higher failure rates [28]; (3) early weight bearing before osseointegration appears to increase fracture risk [11]; and (4) chemotherapy timing may delay osseointegration but does not appear to increase fracture risk once integration occurs [40].

### Clinical interpretation of identified risk factors

These risk factors should be interpreted cautiously because they were derived from small and heterogeneous observational cohorts, often without consistent definitions or adjustment for confounding factors. Nevertheless, the available evidence suggests that device- and treatment-related factors may be more important than demographic variables in determining fracture risk after compressive osseointegration.

### Limitations

This study has some limitations. The small number of patient cohorts in individual studies limited our ability to detect risk factors. The heterogeneity of patient populations (oncological vs. traumatic vs. revision indications) makes direct comparisons challenging. Additionally, the learning curve associated with the technique may have influenced the reported complication rates, particularly in earlier studies. Long-term data beyond 10 years remain limited, and comparative studies directly contrasting the CPS device with traditional cemented stems using matched patient populations are lacking. Most data are from high-volume oncologic centers, which may limit their generalizability to broader orthopedic practice. In addition, the included studies were predominantly retrospective and of moderate methodological quality, which should be considered when interpreting the findings.

## Conclusions

The Zimmer Biomet CPS device appears to have a favorable safety profile, with reported adult periprosthetic fracture rates of approximately 2.7–3.9%. The reported fractures exhibited a clear temporal pattern, occurring predominantly within the first 2 years after implantation, rather than following the progressive failure pattern typically described for traditional cemented stems. Importantly, the bone–implant interface generally remained stable despite fracture, and the short intramedullary footprint of the device may facilitate revision while preserving bone stock. In the pediatric population, although expandable components were associated with a higher overall complication rate, spindle survivorship was 86.3% at 5 years and 66.2% at 10 years, supporting the durability of the compressive osseointegration mechanism. Overall, the CPS device may represent a valuable option for limb salvage reconstruction when careful patient selection, appropriate surgical technique, and strict adherence to postoperative weight-bearing protocols are maintained. Future studies should include longer follow-up, direct comparative analyses using matched populations, and more rigorous evaluation of risk factors and methodological quality.

## Data Availability

No datasets were generated or analysed during the current study.
